# Case Report of Two Cases of Fever, Rash, and Organ Involvement during the Treatment of Leprosy

**DOI:** 10.1371/journal.pntd.0003130

**Published:** 2014-08-28

**Authors:** Hong Liu, Chuan Wang, Tongsheng Chu, Parimi Leela Rani, Debao Yu, Xi'an Fu, Mingfei Chen, Shumin Chen, Furen Zhang

**Affiliations:** 1 Shandong Provincial Institute of Dermatology and Venereology, Shandong Academy of Medical Sciences, Jinan, Shandong, China; 2 Shandong Provincial Hospital for Skin Diseases, Shandong University, Jinan, Shandong, China; 3 Shandong Provincial Key Lab for Dermatovenereology, Jinan, Shandong, China; 4 Shandong Provincial Medical Center for Dermatovenereology, Jinan, Shandong, China; 5 Jinan Institute of Dermatology, Jinan, Shandong, China; CDC, United States of America

## Presentation of Cases

**Figure 1:**
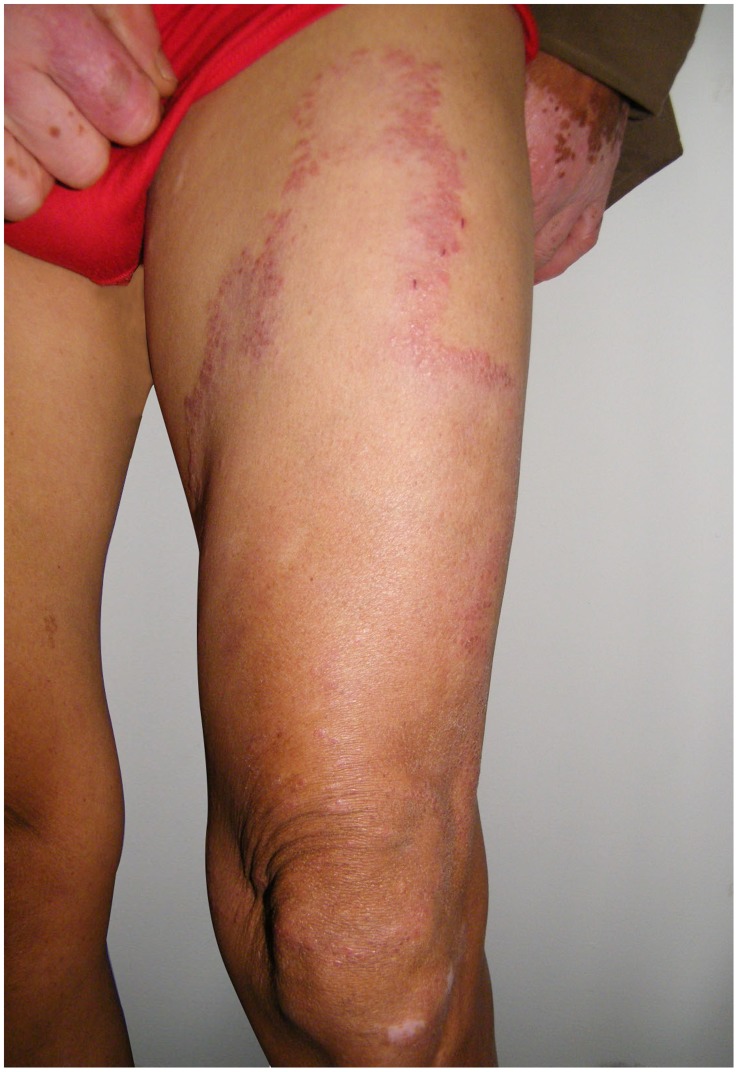
Clinical manifestations of the first patient when he was admitted in our clinics for the first time. Erythema and papules could be observed on the right leg with clear margin.

**Figure 2:**
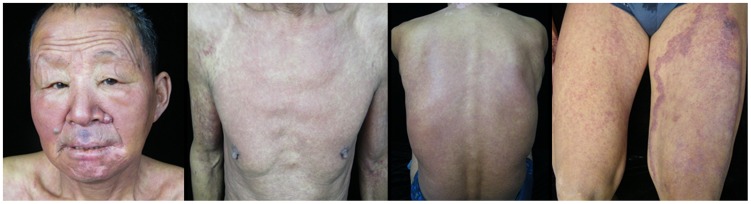
Clinical manifestations of the first patient when he suffered DDS syndrome. Generalized purpuric rash could be found on the face and trunk, and the lesions on the right leg were aggravated.

The first patient was a 65-year-old man whose chief complaint was high fever accompanied by generalized rash for two days. The patient first appeared in the clinics of Shandong Provincial Institute of Dermatology and Venereology with a two-year history of rash (erythema and patches) on his legs and hands without subjective syndromes (Figure 1). Besides the skin, ulnar nerves were found to be thick. Acid-fast bacilli (1+) were found in the tissue smear and histopathological examinations. The diagnosis of borderline tuberculoid (BT) leprosy was confirmed by real-time PCR for *Mycobacterium leprae* gene encoding antigen 85B in the skin samples. Then, multidrug therapy (MDT) (for paucibacillary leprosy: dapsone [DDS] and rifampicin) was given according to the WHO guidelines. This patient was in a good condition for the initial 27 days of treatment until two days before his second admission, when he presented with high-grade fever associated with generalized purpuric rash. Upon hospital admission, his temperature was 39.6°C and inguinal lymph nodes appeared swollen and were painful. Extensive purpuric maculopapular exanthematous rash on the face, trunk and extremities were observed, and the original existing rash on his legs became aggravated (Figure 2). The other system examinations were unremarkable. The patient suffered vitiligo with a history of four years.

The main abnormal laboratory data on admission included the following: white blood cell (WBC), 16.34×10^−9^/L without atypical lymphocytosis or eosinophilia (reference value [RV] is 4.0×10^−9^/L–10.0×10^−9^/L); urine protein (positive); urine bilirubin (positive); urine bilinogen (positive); serum aspartate aminotransferase, 1047 U/L (RV≤41 U/L); alanine aminotransferase, 524 U/L (RV≤42 U/L); gamma-glutamyl transpeptidase, 93 U/L (RV≤40 U/L); alkaline phosphatase, 161 U/L (RV≤117 IU/L); direct bilirubin, 19.9 umol/L (RV≤6.8 umol/L); total bilirubin, 34.2 umol/L (RV≤21 umol/L). Enlargement of inguinal lymph nodes could be observed under high frequency ultrasound.

**Figure 3:**
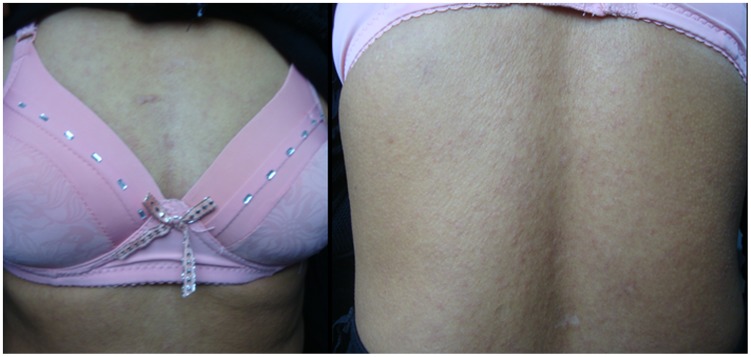
Clinical manifestations of the second patient when she was admitted for the first time. Intensive popular exanthematous rash was found on her trunk.

The second patientwas a 39-year-old woman who was from Yunnan province, an epidemic area of leprosy in China. Her chief complaint was also fever and extensive rash on her trunk for one day. Her first admission was due to the painless ulceration on her feet with a three-year history. Also, ulnar nerves were affected. The examinations of smear test and pathology showed vast acid-fast bailli (5+), and real-time PCR for *Mycobacterium leprae* gene revealed the specificity of bacilli, which supported the diagnosis of lepromatous leprosy. MDT for multibacillary leprosy (DDS, clofazimine, and rifampicin) was given accordingly. She was readmitted 21 days after she underwent MDT, with presentation of fever and generalized pruritic rash. Upon admission, her temperature was 39.2°C and pruritic intensive papular exanthematous rash on the trunk and extremities were observed (Figure 3). The other system examinations were normal.

The laboratory investigation revealed that hemoglobin was at 74 g/L (RV≥110 g/L) with signs of haemolysis, while the value of her Glucose-6-phosphate dehydrogenase was normal. Mild elevation of alanine aminotransferase, 48 U/L (RV≤41 U/L) was found. Other indicators were normal.

## What Is the Diagnosis and the Differential Diagnosis?

All the symptoms of the two patients were firstly suspected to be a drug-induced hypersensitivity syndrome (DIHS) caused by MDT, especially DDS. Of course, rifampicin was also reported to be one of sensitizing drugs of DIHS [1]–[4]. We could not exclude the diagnosis of leprosy reactions totally at this phase.

While treating leprosy patients, physicians should be well familiarized with conditions such as DIHS and leprosy reactions. Due to the distinct managements, timely differentiation of the two diseases is important. The former usually develops within two months after drug introduction and presents clinically as an extensive rash, accompanied by fever and the involvement of organs, such as lymphadenopathy, hepatitis, hematologic abnormalities and so on. Leprosy reactions include two types: Type 1 reaction (T1R), or reversal reaction, and type 2 reaction (T2R), or erythema nodusum leprosum (ENL). T1R and T2R may occur separately or together, or one after the other, and may appear before, during, or after the treatment of leprosy. In T1R, skin involvement (new lesions may suddenly appear and/or old lesions become red and swollen) frequently accompanies nerve involvement. T2R is a generalized disease, which could affect skin, nerves, and other organs such as joints and lymph nodes, often accompanied by fever and elevated WBC count. Sometimes it is difficult to distinguish DIHS from T1R and T2R, especially when the symptoms are not complete in these two diseases.

For the first patient, with an elevated WBC of 16.34×10^−9^/L and high fever of 39.6°C, T2R was not taken into account because T2R typically occurs in the patients who undergo treatment for lepromatous or borderline lepromatous forms of leprosy, not in BT patients. Also, in these two patients there were no complaints of nerve pain and no significant signs of nerve damage were observed under ultrasound. High-grade fever and rash were the first signs after drug intake. All the above findings inclined towards the diagnosis of DIHS. There were more reported cases of DDS being the offending agent in DIHS rather than rifampicin. So, we speculated that DDS induced this DIHS.

## What Led to the Final Diagnosis of Dapsone Induced Hypersensitivity Syndrome?

To find more evidence for diagnosis and to decide whether DDS or rifampicin induced the hypersensitivity reactions, we performed an analysis using HLA-B*13:01 test in the two patients. HLA-B*13:01 has been identified as a risk predictor of DDS-induced hypersensitivity syndrome (DDS syndrome) with 85.5% of sensitivity and 85.7% of specificity [5]. The presence or absence of the HLA-B*13:01 risk allele was determined using sequence-specific polymerase-chain-reaction assay primers (PCR-SSP), and the electrophoresis results of the two cases showed both of the patients carried one risk allele of HLA-B*13:01, which supported the diagnosis of DDS syndrome.

**Figure 4:**
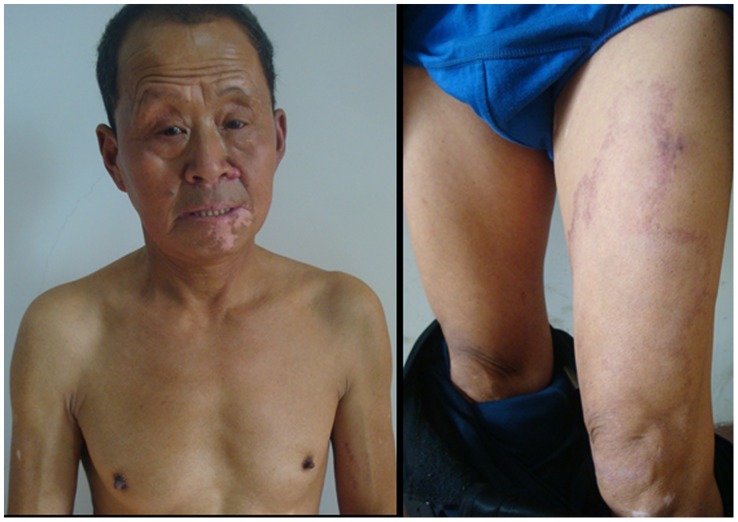
Clinical manifestations of the first patient after the treatment. The rash of DDS syndrome disappeared and only original lesions could be found on his leg.

**Figure 5:**
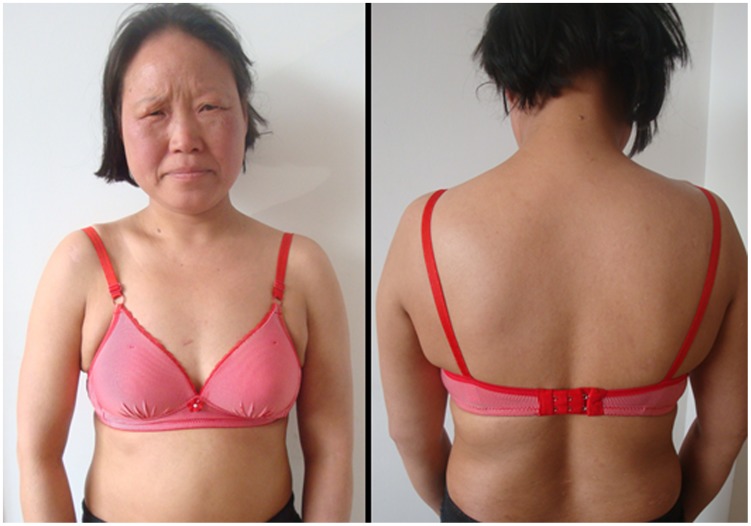
Clinical manifestations of the second patient after the treatment. The rash disappeared totally.

To be on the safe side, MDT was stopped immediately and corticosteroids were given orally (prednisolone 1 mg/kg/d). Their clinical condition improved quickly, and laboratory test results returned to normal levels within about one month (Figure 4 and 5). Then the steroids were slowly tapered and stopped over a period of nearly seven weeks.

After that, DDS was removed from MDT. For the first patient, rifampicin and clofazimine as an alternative drug combination were used for leprosy treatment, and no recurrence of DDS syndrome was observed during the follow-up of six months. For the second patient, also, rifampicin and clofazimine were used in treatment. However, she suffered another attack of fever and rash, as she mistakenly took one pill of DDS. After being treated with prednisolone again for one month, all the clinical symptoms disappeared and no recurrence of DDS syndrome was observed in the follow-up of three months.

Our diagnosis of DDS syndrome was based on the combination of symptoms, the two patients' medical history, and the results of HLA-B*13:01 test. The two patients remain under supervised treatment for leprosy, with good outcome.

## What Is the Difference between Complete DDS Syndrome and the Incomplete Form?

The first patient presented the full symptoms, including fever, lymphadenopathy, generalized rash, and abnormal liver function after DDS intake, which met the criteria of complete DDS syndrome proposed by Richardus and Smith [6]. But not all those features are necessarily needed to be present at the same time. In some review studies of DDS syndrome, only 59% of cases could be classified as complete DDS syndrome forms [7]. The second patient only presented fever, extensive rash, and mild elevated alanine aminotransferase, and based on her medical history and the result of HLA-B*13:01 test, the diagnosis of DDS syndrome was confirmed. Here we can infer that timely admission and appropriate treatment might have prevented the involvement of other organs.

## What Is the Clinical Value of the HLA-B*13:01 Test?

We performed a HLA-B*13:01 test in the two cases, which provided additional evidence to support the diagnosis of DDS syndrome. What's more, this test makes it possible to identity those individuals at risk for DDS syndrome and thus helps to improve the safety of DDS therapy. Up to now, the HLA-B*13:01 test was performed in 33 new leprosy patients and six new dermatitis herpetiformis patients in our clinics before treatment using MDT or DDS alone. Among them, only one leprosy patient carried one risk allele of HLA-B*13:01 and in that case, DDS was removed from MDT. All the patients were followed up for 3 months and no one suffered DDS syndrome.

To summarize, identifying the carriers of HLA-B*13:01 allele and modifying their therapeutic regimen can greatly lower or eliminate the risk of DDS syndrome.

## Case Discussion

DDS syndrome is a life-threatening condition in the treatment of leprosy with an incidence of 0.5%–3.6% [8]–[10] and an associated mortality of 9.9% [11]. Timely and correct diagnosis and differential diagnosis is the key to further management. Physicians, mainly in geographical areas with high prevalence rates of leprosy, should be aware of this potentially fatal condition.

Recently, more and more risk markers of drug-induced reactions have been identified, making prediction of a reaction before drug therapy possible, which has led to an evident decrease in the incidence of some drug-induced reactions [12], [13]. In addition to its application in the diagnosis and differential diagnosis of DDS syndrome, the HLA-B*13:01 test should be conducted before DDS intake, so that practitioners can prevent this condition by removing DDS from MDT for those who carry the HLA-B*13:01 risk allele.

**Key Learning Points**

- This case report is a useful reminder that clinicians should be aware of the possible fatal adverse effects of dapsone (DDS).
- The HLA-B*13:01 test is useful to distinguish DDS syndrome from leprosy reactions.
- The HLA-B*13:01 test before treatment will be useful to prevent DDS syndrome.
